# Electrochemical performance and microbial community profiles in microbial fuel cells in relation to electron transfer mechanisms

**DOI:** 10.1186/s12866-017-1115-2

**Published:** 2017-10-18

**Authors:** Naroa Uria, Isabel Ferrera, Jordi Mas

**Affiliations:** 1 Departament de Micro-Nano Sistemes, Institut de Microelectrònica de Barcelona (IMB-CNM), CSIC, Bellaterra, Spain; 2 Departament de Genètica i Microbiologia, Universitat Autònoma de Barcelona, Bellaterra, Spain; 3 Departament de Biologia Marina i Oceanografia, Institut de Ciències del Mar (ICM), CSIC, Barcelona, Spain

**Keywords:** Microbial fuel cell, Electrogenic bacteria, Electron transfer, Pyrosequencing

## Abstract

**Background:**

Microbial fuel cells (MFCs) operating with complex microbial communities have been extensively reported in the past, and are commonly used in applications such as wastewater treatment, bioremediation or in-situ powering of environmental sensors. However, our knowledge on how the composition of the microbial community and the different types of electron transfer to the anode affect the performance of these bioelectrochemical systems is far from complete. To fill this gap of knowledge, we designed a set of three MFCs with different constrains limiting direct and mediated electron transfer to the anode.

**Results:**

The results obtained indicate that MFCs with a naked anode on which a biofilm was allowed unrestricted development (MFC-A) had the most diverse archaeal and bacterial community, and offered the best performance. In this MFC both, direct and mediated electron transfer, occurred simultaneously, but direct electron transfer was the predominant mechanism. Microbial fuel cells in which the anode was enclosed in a dialysis membrane and biofilm was not allowed to develop (MFC-D), had a much lower power output (about 60% lower), and a prevalence of dissolved redox species that acted as putative electron shuttles. In the anolyte of this MFC, *Arcobacter* and *Methanosaeta* were the prevalent bacteria and archaea respectively. In the third MFC, in which the anode had been covered by a cation selective nafion membrane (MFC-N), power output decreased a further 5% (95% less than MFC-A). In this MFC, conventional organic electron shuttles could not operate and the low power output obtained was presumably attributed to fermentation end-products produced by some of the organisms present in the anolyte, probably *Pseudomonas* or *Methanosaeta.*

**Conclusion:**

Electron transfer mechanisms have an impact on the development of different microbial communities and in turn on MFC performance. Although a stable current was achieved in all cases, direct electron transfer MFC showed the best performance concluding that biofilms are the major contributors to current production in MFCs. Characterization of the complex microbial assemblages in these systems may help us to unveil new electrogenic microorganisms and improve our understanding on their role to the functioning of MFCs.

**Electronic supplementary material:**

The online version of this article (10.1186/s12866-017-1115-2) contains supplementary material, which is available to authorized users.

## Background

A microbial fuel cell (MFC) can be defined as a bioelectrochemical system in which microorganisms act as catalysts to convert chemical energy into electrical energy. Microbes in the anode chamber oxidize reduced substrates generating electrons and protons in the process. Unlike in aerobic metabolism, electrons are absorbed by the anode, acting as an artificial electron acceptor and then, transported to the cathode through an external circuit. So far, the most immediate and useful applications of MFCs are related to wastewater treatment and bioremediation, and to environmental sensors power supply (for example, sediment microbial fuel cells). In any case, the microbial component is crucial on the performance of the process. These applications are often run using complex microbial communities that are developed spontaneously in the MFC anode from inocula of different origins, generating higher power densities than pure cultures [1].

A relative large number of studies have described the bacterial communities of MFCs operating under different conditions (inoculum, carbon source, anodic potential, external resistance or pH) [2–4]. Different classes of Proteobacteria, Firmicutes and Acidobacteria phyla have shown the ability of generating electrical current. Likewise, some eukaryotes such as some microalgae, yeast and fungi have also been reported growing in these systems [5]. However, the links between the microbe’s identity and their electrogenic activity and, therefore, their capacity of current production and contribution to MFC performance, are barely known. The complexity of the bacterial interactions within the community and the fact that different electron transfer mechanisms may be used by the same organism, makes it difficult to determine or analyze the transfer processes and therefore, to determine their role in current production.

As a rule, the different mechanisms proposed to explain the electron transfer to the anode in MFCs are classified depending on whether electron transfer to the electrode surface occurs through soluble compounds (mediated electron transfer) or through bacterial membrane redox active proteins and conductive pili (direct electron transfer) [5, 6]. Mediated electron transfer (MET) is carried out by organic redox species of microbial origin secreted to the medium. These redox species are able to accept electrons from the cellular electron transport chains, and diffuse to the electrode surface where they are reoxidized to their initial state, thus effectively acting as electron shuttles between cells and the electrode [7]. In MFCs operating with complex communities, shuttles excreted by one organism can be taken up and used by another organism for a large number of redox cycles [8]. Likewise, although at low efficiency due to their slow reaction with the electrode [9], some metabolic end-products produced by fermentation can also be oxidized at the electrode surface producing electric current [7]. Contrarily, direct electron transfer (DET) proceeds via membrane-bound redox proteins. It has been proved that some microorganisms have redox active proteins, such as *c*-type cytochromes and iron sulfur proteins, localized to the outer-membrane that are able to perform the electron transfer to solid-phase electron acceptors [6, 10]. Additionally, some microorganisms can exchange electrons through electrically conductive protein filaments called “nanowires” [6, 11, 12].

While it is well known that this mechanism coexist in microbial fuel cells harboring complex microbial communities, the extent to which each of these mechanisms contributes to microbial fuel cell operation has not been determined. In this work, we attempt to gain insights on the electrogenic capacity of the organisms present in complex communities while determining the influence of the different electron transfer mechanisms on MFC performance. To this end, different anode configurations that restricted the operation of one or more of the electron transfer mechanisms mentioned above were used. In one of them, MFC-A, unrestricted biofilm development is allowed on the surface of a naked anode and all electron transfer mechanisms can operate. In a second configuration, MFC-D, the anode is enclosed in a dialysis membrane and direct electron transfer cannot occur. The third configuration, MFC-N, uses a nafion-covered that restricts most organic electron shuttles from reaching the anode while allowing unrestricted movement of protons and gases. Comparison of the power output generated by each setup allows us to draw conclusions regarding the relative contribution of the different electron transfer mechanisms to overall MFC performance.

Using these controlled setups, the microbial community developed can be simplified and studied in reference to the electron transfer mechanisms and MFC. In addition, by using acetate as the only carbon source, we simplify the metabolic landscape that could emerge when using more complex carbon sources. To obtain comprehensive information on microbial composition, high-throughput sequencing was applied to characterize anodic biofilms and suspended community samples. The study of the microbial communities developed in each of the three MFC together with their electrochemical performance enables us to better understand the role of some of these common anode bacteria in current production as well as the contribution of the different electron transfer mechanisms. We anticipate that this knowledge can be of great importance to enhance MFCs performance and their applications.

## Methods

### MFC design and set-up

In this study, three two-chamber fuel cells of a 1 Lvolume were used. Nafion®117 (Ion power, Inc.) was used as proton exchange membrane (PEM) with a thickness of 183 μm and effective area of 11.34 cm^2^. The three MFCs were operated with gentle stirring in a temperature controlled room at 28 °C. The cathode and anode chambers were gently sparged with air and nitrogen, respectively, except during periods of headspace gas sampling. In all cases the cathode chamber contained 0.1 M phosphate buffer. Cathodes consisted of 75.91cm^2^ silicon wafers coated with platinum manufactured as previously described [13].

The anode chambers were filled with 800 mL of AB minimal medium [14], containing acetate (10 mM) as carbon source. A volume of 10 mL of sediment-water slurry obtained from a small creek running through the campus of the Autonomous University of Barcelona (Spain) was added to each reactor as inoculum. Following inoculation, the MFCs were operated in batch mode for a period of 31 days with sequential feeds of acetate at day 7 and day 23, immediately after sampling.

Three different MFC experiments with different anode configurations were set up: (i) MFC-A with a naked anode, (ii) MFC-D with an electrode covered by a dialysis bag, and (iii) MFC-N with a nafion coated electrode. In all cases, the anodes were made of carbon paper (B2120 Toray Carbon Paper Designation TGPH-120, plain, E-Tek, Inc.) with a thickness of 0.35 mm and an area of 10 cm^2^. First, MFC-A had a naked carbon paper anode that allowed both soluble redox shuttles and bacteria to contribute to current production. In the second cell, MFC-D, the carbon paper anode was enclosed in a dialysis bag previously autoclaved and filled with AB minimal medium. The use of the dialysis bag prevented bacteria from passing through, while soluble molecules were able to reach the anode. The goal of this set up was to estimate the contribution of mediator-dependent processes to current generation and power output. The dialysis bag employed was a Spectra/Por® 3 Regenerated Cellulose membrane (SpectrumLabs), which had a molecular weight cut off rating of 3.5 Da. Finally, in the third cell, MFC-N, the carbon paper anode was coated with 4 layers of Nafion ion exchange resin (20 wt.% soln. in lower aliphatic alcohols/H_2_O, Sigma-Aldrich Co.). Since nafion only allows cations and gases to reach the anode, we expect neither direct contact nor conventional redox shuttles to be operative, thus, severely limiting both direct and mediated electron transfer mechanisms.

### Microbial fuel cell operation and characterization

MFCs were operated under an external load of 65 KΩ. Voltage across this load was continuously monitored, using a digital multimeter data acquisition system (HP 34970A, Agilent, USA). Polarization curves were recorded daily using a source meter unit Keithley®2612 (Keithley Instruments Inc., USA). Power and current values were normalized to the anode area. Likewise, internal resistance (R_int_) was calculated from the slope of the polarization curves using:

1 $$ U= OCV-j\cdotp R\operatorname{int}, $$

being: U, MFC voltage in a determinate current intensity; OCV, the open circuit potential; j, the current density; and, R_int_, the internal resistance [15].

Anode and cathode working potentials were measured during operation under the external resistance mentioned above using a Fluke 112 True RMS Multimeter (Fluke Corporation, Everett, WA) and an Ag/AgCl reference electrode. All potentials have been expressed referred to a standard hydrogen electrode (SHE).

### Electrochemical characterization of anolytes and anodes

Cyclic voltammetries of anolytes and anodes were performed using a potentiostat/galvanostat model FRA2 Micro-Autolab Type II. An Ag/AgCl electrode (Methrom, Switzerland) and a platinized silicon wafer electrode were used as reference and auxiliary electrode, respectively. Cyclic voltammetries were run between −0.7 V and 0.4 V (vs. Ag/AgCl) at a scan rate of 1 mV·s^−1^.

Analysis of the redox species present in the different anolytes was conducted at different times during the experiment. Ten mL of anode solution were filtered using a sterile filter with a pore size of 0.22 μm (Millex**®**GP, Millipore) to remove bacteria, and introduced in an electrochemical cell. A carbon paper electrode with an area of 0.5 cm^2^ was used as working electrode.

In order to perform voltammetries of the biofilm, the anodes of the each MFC were removed from the reactor at the end of the experiment. After washing several times in phosphate buffer, these electrodes were then used as working electrodes submerged in 100 mL of oxygen free phosphate buffer (0.1 M).

Cyclic voltammetry analyses of the electrodes and medium before inoculation were also performed as blanks. No peaks were observed in any case.

In all cases, the electrolyte solution was flushed with nitrogen to remove oxygen traces before conducting the CV analyses. For the sake of comparison, current values were normalized to the area of working electrodes. Potentials were also expressed vs. SHE.

### Chemical analyses

Approximately 4 times a week, 1 mL of anode solution of each MFC was collected and filtered, using a sterile filter with a pore size of 0.22 μm (Millex**®**GP, Millipore) in order to determine the concentration of acetate. Samples were analyzed by gas chromatography using a 7820A Agilent GC System (Agilent, USA) with helium as carrier gas and a flame ionization detector.

Gas production in the reactors was monitored using 1 L polypropylene sample bags (245-2× Series Sample Bag, SKC Inc., USA) connected to the anode chambers headspaces. The contents of the bags were analyzed weekly in a gas chromatograph with argon as carrier gas using a thermal conductivity detector (TCD).

### Biomass determination

For epifluorescence microscopy counts, samples were fixed with formaldehyde (0.4% final concentration) (Sigma, USA) and filtered through 0.2 μm pore size polycarbonate GTBP filters (Millipore, USA). Bacteria were stained with 4′-6′-diamidino-2-phenylindole (DAPI) (Merk, Germany) for 5 min and then, rinsed in phosphate buffered saline (PBS) immediately prior to imaging. The filters were mounted on glass slides using immersion oil and were visualized in a Zeiss AXIO Imager A1 fluorescence microscope (Zeiss, Germany).

### Biofilm morphology and viability

Viability of cells in the biofilm on MFC-A electrode and the presence of cells on MFC-D and MFC-N was determined using the bacterial viability test Live/Dead®BacLight™ Bacterial Viability Kit (Invitrogen) by following the protocol detailed by the supplier. Images were acquired with a Zeiss AXIO Imager A1 fluorescence microscope (Zeiss, Germany).

Scanning Electron Microscopy (SEM) and Confocal Laser Scanning Microscopy (CLSM) images were obtained from the biofilm formed at MFC-A since the modification of the MFC-D and MFC-N electrodes prevented the formation of the biofilm as it was observed by fluorescence microscopy. Before staining, the electrode was washed in phosphate buffer and cut in different fragments of 0.5 cm^2^. Scanning electron micrographs were taken using an EVO®MA 10 microscope (Zeiss, Germany) after the electrode fragment was coated with gold.

For CLSM, the electrode samples were prepared with HOETSCH DNA stain that allows observation of bacterial cells, while exopolymers in the biofilm were visualized with Alexa Fluor®488 conjugated Concavalin A (ConA) and Alexa Fluor®594 conjugated wheat germ agglutinin (WGA). Images were obtained with a confocal microscope Leica TCS SP2 AOBS (Leica, Germany).

### Community analysis

#### DNA extraction and amplification of 16S rRNA genes

At 0, 8, 15, 29 and 31 days after the start of operation, 50 mL samples of each anolyte were filtered onto 0.2 μm polycarbonate filters (Millipore GTTP4700) and filters stored at −20 °C until further analyses. At the end of the experiments, DNA was extracted from the filters using the UltraClean water kit (MOBIO ref. 14,880–25). Additionally, after 31 days of operation, the anodes of MFC-A and MFC-N were subjected to DNA extraction using the PowerSoil kit (MOBIO ref. 12,888–50). DNA integrity was checked by agarose gel electrophoresis and quantified by absorbance measurements at a wavelength of 260 nm.

#### Sequence generation and analyses

Before sequence generation, we investigated the stability of the microbial communities over time by using Denaturing Gradient Gel Electrophoresis (DGGE) [16–19]. Based on DGGE results (data not shown) and the chemical data available, we selected a sampling time to characterize the communities in depth by applying high-throughout sequencing. Pyrosequencing of 16S rRNA gene was performed by the Research and Testing Laboratory (Lubbock, TX, USA; http://rtlgenomics.com/) using the bacterial Tag-Encoded FLX Amplicon Pyrosequencing (bTEFAP) method as described previously [20]. Primers 28F (5′- GAGTTTGATCNTGGCTCAG-3′) and 519R (5′- GTNTTACNGCGGCKGCTG-3′) generated amplicons spanning the V1 to V3 regions of the bacterial 16S rRNA gene, and primers 341F (5′-GYGCASCAGKCGMGAAW-3′) and 958R (5′-GGACTACVSGGGTATCTAAT-3′) were used to amplify archaeal fragments spanning the V3 to V5 regions. The generated pyrosequencing data were processed using the QIIME (Quantitative Insights Into Microbial Ecology) pipeline as described in Ferrera et al. [21]. Briefly, a sequence filtration step was performed before denoising sequences to reduce the impact of pyrosequencing errors. Curated sequences were grouped into operational taxonomic units (OTUs) or phylotypes with a minimum identity of 97%. A representative sequence from each phylotype was chosen by selecting the most abundant sequence in each cluster. The resulting representative sequences were checked for chimeras and the identity of 16S rRNA phylotypes was determined using the RDP Classifier implemented in QIIME. Chao1 diversity metrics and rarefaction curves were computed in QIIME and plotted in Kaleidagraph (v.4.1). Shannon diversity index (H´) was calculated as the sum of the proportion of a certain species multiplied by its natural logarithm as explained by Magurran [22]:

2 $$ {H}^{\prime }={\sum}_{i=1}^{i=n} pi\  Ln\  pi, $$

Where *n* is the number of species in a sample (number of OTUs) and *p*_*i*_ the proportion of a certain species (number of sequences).

Differences in microbial community structure were visualized by principal component analysis calculated based on weighted Unifrac metrics using QIIME. Sequence data have been deposited in the MG-RAST public database (http://metagenomics.anl.gov/) under project “Microbial Fuel Cell” with the number: MFC (4,690,147.3).

## Results

### Acetate consumption and microbial growth

**Fig. 1:**
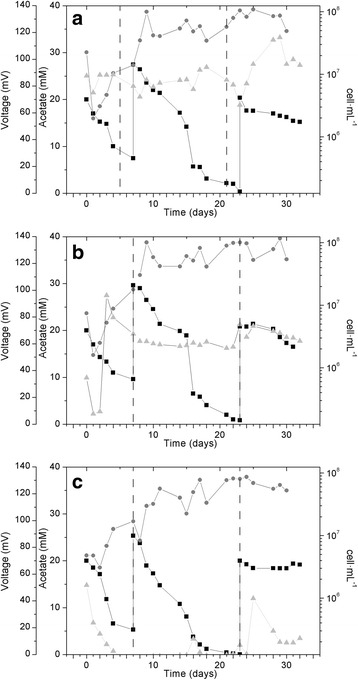
Evolution of acetate (■), biomass concentration (●), and cell voltage (▲) under operational conditions (65 KΩ) along the experiment in MFC-A (**a**), MFC-D (**b**) and MFC-N (**c**). Dashed lines show the acetate feeds

Biomass measurements showed a steady increase in the three MFCs during the first 10 days of the experiment, stabilizing thereafter (Fig. 1). The fluctuations in biomass concentration under the three reactor conditions were not significant (*p* > 0.05). At the beginning of the experiment, cell densities in the anolyte of the three MFCs averaged 1.16x10^7^cells·mL^−1^. During the first week, cell numbers increased steadily up to a maximum values of 1.09 × 10^8^, 1.17 × 10^8^ and 8.65 × 10^7^ cells·mL^−1^ for MFC-A, MFC-D and MFC-N, respectively. This increase in cell density was accompanied by a decrease in acetate. Before the first addition, 60% of the acetate had been consumed. For the second addition, acetate was fed when concentration fell below 5% of initial value. After a second addition of acetate, cell concentration barely increased, and acetate concentration remained almost unchanged, indicating that the stabilization of bacterial growth was not due to a limitation in the carbon source but to a depletion of some other essential compound. SEM and CLSM were used to visualize the structure and to determine the thickness of the biofilm formed on the anode of MFC-A, since this was the MFC in which biofilm had a role in current production. CSML images of the electrode biofilm showed an average biofilm thickness of 55.6 ± 1 μm. Additionally, biofilm on the anode electrode was also observed by fluorescence microscope using Live/Dead staining, revealing that most of the cells in the biofilm were alive (data not shown).

Methane and hydrogen production were measured to detect fermentation processes (see Methods). Traces of methane were found in MFC-N only during the first week. These results indicate that methanogenesis was likely not occurring in any of the three MFCs. Furthermore, hydrogen was not detected in any of the three MFCs.

### Electrical performance

MFCs were run under the same conditions for 4 weeks. Voltage (U) was continuously recorded and both, anode and cathode potentials, were measured under these conditions. After stabilization, average voltages of 90.9 ± 1.8, 64.6 ± 3.8 and 11.28 ± 1.7 mV were measured in MFC-A, MFC-D and MFC-N, respectively, showing the MFC with non-coated anode (MFC-A) the best performance (Fig. 1, Table 1).

**Table 1:** Summary of the results obtained for the three experimental setups, as average values ± standard errors. Data were obtained after stabilization by continuous operation at 65 KΩ and by the polarization and power curves made

	MFC-A	MFC-D	MFC-N
U/mV (65 KΩ)	90.9 ± 1.8	64.6 ± 3.8	11.28 ± 1.7
OCV/mV	144.9 ± 5.33	132.2 ± 7.85	34.04 ± 8.67
R_int_/KΩ	43.7 ± 2.7	78.1 ± 2.17	54.49 ± 5.63
Max *j*/μA·cm^−2^	0.34 ± 0.04	0.16 ± 0.01	0.06 ± 0.01
Max P_d_/μW·cm^−2^	1.0e^−2^ ± 1.2e^−3^	4.99e^−3^ ± 4.08e^−4^	5.21e^−4^ ± 2.7e^−4^
E° Cathode/V (vs SHE)	0.18 ± 0.005	0.20 ± 0.002	0.28 ± 0.02
E° Anode/V (vs SHE)	−0.03 ± 0.01	−0.05 ± 0.005	−0.23 ± 0.013

From the start of the experiment, MFC-A showed the highest anode potentials with an average of −0.03 ± 0.01 V. Initial anode potentials (E° anode) of MFC-D and MFC-N started from lower values likely as a result of the impossibility of certain redox species to reach the electrode. MFC-D anode potential suffered a slow increase during the first five days probably due to an increase in the oxidation of redox species in the anode, until reaching values similar to those of the MFC-A with an average of −0.05 ± 0.005 V. MFC-N anode potential showed the lowest values of the three reactors (−0.23 ± 0.013 V), indicating a higher difficulty to electron transfer to the anode and the accumulation of even higher amounts of reduced compounds. Cathode potentials (E° cathode) registered ranged between 0.18 ± 0.005 V and 0.28 ± 0.02 V (Table 1) being stable and similar in all MFCs, indicating that the anode seemed to be the limiting factor for voltage output.

**Fig. 2:**
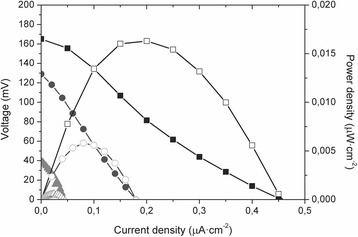
Polarization (solid symbols) and power density (open symbols) curves of MFC-A (■), MFC-D(•) and MFC-N (▲) at the end of the experiment

Polarization curves obtained daily showed that the three MFCs had different power outputs. Average values after stabilization of OCV, maximum power density (Max P_d_) and maximum current density (Max *j*) obtained from polarization curves are listed in Table 1. Additionally, Fig. 2 shows the curves obtained at the end of the experiment. As expected, the non-coated anode MFC-A, showed the best performance with higher OCV and Max *j* values (Table 1). Although MFC-D showed similar OCV values to MFC-A, the Max *j* values were about 50% lower. Finally, the MFC-N displayed the lowest values in all parameters as compared to the other MFCs reflecting the fact that neither bacteria nor conventional electron shuttles could react with the electrode.

### Electrochemical measurements

**Fig. 3:**
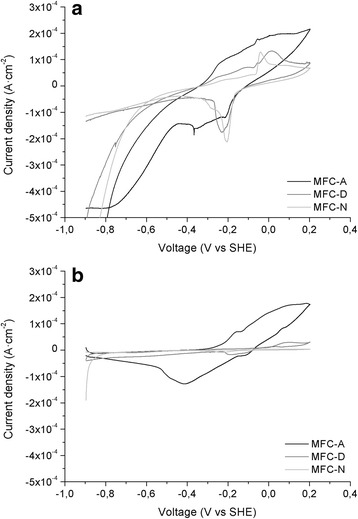
Cyclic voltammograms at a scan rate of 1 mV·sec^−1^ of anolytes (**a**) and anode electrodes (**b**) at the end of the experiment

After removing cells in suspension by filtration, cyclic voltammetries of the anolytes were run at different times to check for soluble redox compounds. Cyclic voltammetries of the anolyte presented in Fig. 3a indicated the presence of a large number of redox active compounds likely corresponding to soluble redox shuttles (see Additional file 1: Table S1 for details). Cyclic voltammetries of MFC-A and MFC-D anolytes showed several redox peaks, many of them compatible with some of the mediators described in the literature such as 2-amino-3-carboxy-1,4-naphtoquinone (ACNQ) (−0,071 V) [23], pyocianine (−0.03 V) [24], pyrroloquinoline quinone (PQQ) (−0.4 V) [25], and flavins (−0.2/−0.25 V) [26]. Noteworthy, in the anode chamber of MFC-D, where conditions favor the mediated electron transfer, the number of redox species was the highest.

The voltammogram obtained to the MFC-N anolyte showed accumulation of redox compounds in the medium despite these cannot contribute to current production because of the nafion barrier.

Additionally, the anodes were also analyzed by cyclic voltammetry at the end of each experiment (Fig. 3b). Results show that the biofilm grown on the MFC-A electrode contained an electrochemically active community.

Despite the voltammogram for the anode of MFC-D was expected to be close to 0, it also presented a small peak (−0.2 V) coincident with a large peak found in the anolyte. These results suggest that a small quantity of soluble redox active compounds remained adsorbed to the graphite electrode. The voltammogram of the MFC-N anode, as expected, showed no peaks.

### Microbial community analyses

Reactors were operated for 1 month and during this period, stabilization of the microbial community composition was observed by using DGGE (data not shown). 454-pyrosequencing of bacterial and archaeal 16S rRNA gene was used to characterize the stable microbial community associated with anolytes and anode electrodes. Pyrosequencing of all bacterial and most archaeal amplicons was successful. After a rigorous quality check of the sequences (see Methods), a total of 65,204 bacterial (average per sample, 10,867) and 11,573 archaeal (average per sample, 1929) 16S rRNA high-quality sequences were kept in the analyses (Additional file 2: Table S2). Clustering of reads into OTUs at 97% cutoff resulted in a total of 1220 different bacterial and 135 archaeal OTUs (Additional file 2: Table S2).

In general, the number of archaeal reads was lower than the bacterial reads in all samples. Although we cannot rule PCR- and primer-related biases out [27], it is likely that the low number of reads of Archaea is due to lower abundances or even absence in those samples with negative amplification. In fact, the highest number of archaeal reads was obtained in the inoculum rather than in the communities developed in the MFC.

### α- and ß-diversity of MFCs bacteria

Patterns of α- and ß-diversity [22] were assessed based on OTU-based metrics using rarefied OTU tables at even depth (3800 reads which was the lowest value obtained). For α-diversity, calculation of the Chao1 richness estimator (Additional file 4: Fig. S1) showed that the diversity in the samples was probably higher than what could be described since curves were not asymptotic (Additional file 4: Fig. S1). Moreover curves showed that richness was much higher in the inoculum than in the MFC samples. Yet, when comparing the MFC reactors, no clear differences were observed neither in richness (observed OTUs) nor in diversity (as Shannon Weaver index) (data not shown).

**Fig. 4:**
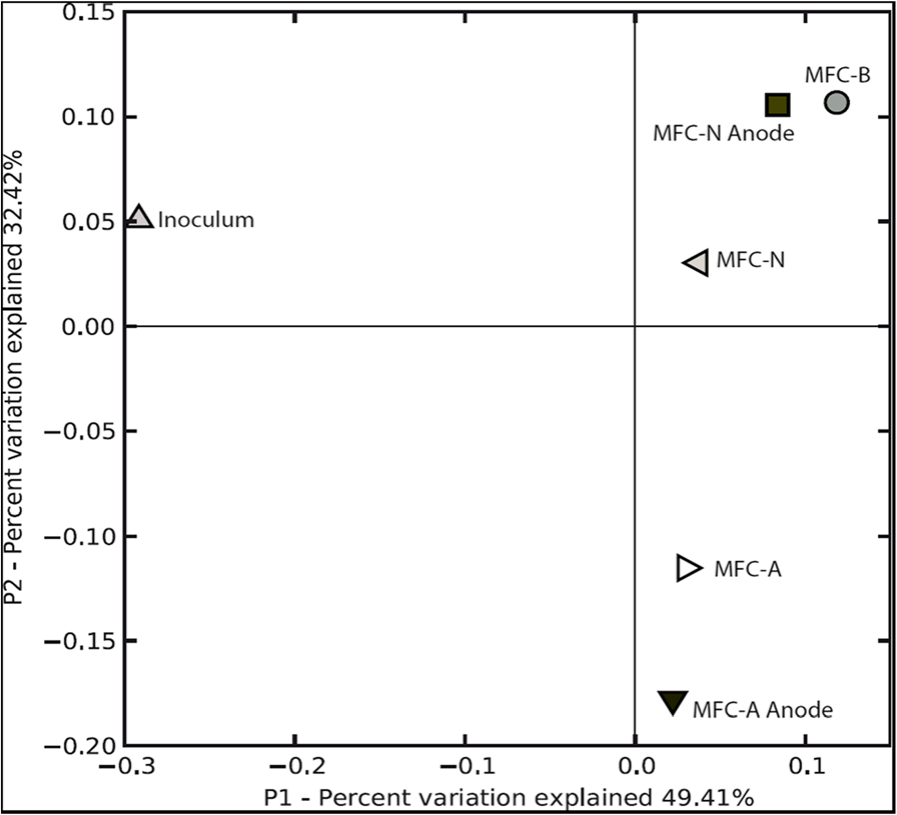
Principal coordinate analysis of the samples based on UniFrac distances. The percentages in the axis labels represent the percentages of variation explained by the principal coordinates

Differences in microbial composition and structure (ß-diversity) were calculated based on weighted Unifrac metrics. The weighted UniFrac is a method that calculates a distance matrix between communities using phylogenetic information while accounting for the relative abundance of each OTU. Principal component analysis showed that all MFC samples clustered separated from the inoculum, indicating that only a few taxa present in the original sample were able to thrive in the MFCs (Fig. 4). Among the MFCs, the anodic biofilm and suspended bacterial community of MFC-A clustered together, MFC-D clustered closer to MFC-N (suspended and biofilm).

### Taxonomy of bacteria

**Fig. 5:**
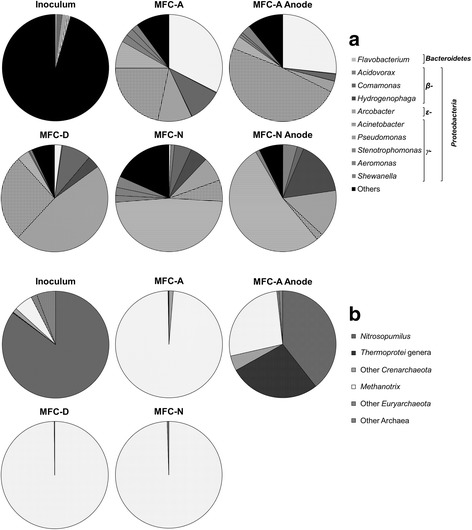
Distribution at the genus level obtained from pyrosequencing reads of (**a**) bacterial and (**b**) archaeal communities. “Others” include genera with abundances lower than 0.1%

To get insights on how MFCs communities differed taxonomically, we assigned an identity to each OTU (Fig. 5a). After the period of MFC operation, the microbial community differed significantly from the inoculum in all cases. Furthermore, clear differences in the genera developed in the three reactors were found and only a small percentage of OTUs was shared between the different MFCs (only 12% were shared in all samples). We found that most sequences in all MFC reactors were related to the phyla Proteobacteria and Bacteroidetes (Fig. 5a, Additional file 3: Table S3). Both phyla were also abundant in the inoculum (~41% and ~9%, respectively) but they were enriched in the MFCs, specially the Proteobacteria, which reached abundances up to 99% of the total bacterial community. Within the Proteobacteria, the most abundant classes were the *γ*- (62.4%), *ε-* (19.3%) and *β-Proteobacteria* (17.6%), but *α-* (0.65%) and *δ-Proteobacteria* (<0.1%) were also present (Additional file 3: Table S3). The Bacteroidetes present belonged primarily to the Flavobacteriales (88.5% of total Bacteroidetes).

In MFC-A, both the suspended and biofilm communities were dominated by the classes *γ-Proteobacteria* and *Flavobacteria* (Additional file 3: Table S3). The most prevalent genera in MFC-A were *Flavobacterium* and *Acinetobacter* with a relative abundance of 26.7% and 48.7%, respectively in the anode, and 32.5% and 21.6% in the anolyte (Additional file 3: Table S3, Fig. 5a). In the case of MFC-D anolyte, despite members of the *γ-Proteobacteria* such *as Pseudomonas, Shewanella* or *Acinetobacter,* were also abundant, the community was largely dominated by the *ε-Proteobacteria,* in particular by *Arcobacter* (47%) (Fig. 5a)*.* Finally, the *γ-Proteobacteria* were also numerous in MFC-N community (65.6%) (Additional file 3: Table S3). Notably, *Pseudomonas* was the most prevalent genus in the anolyte sample, with a relative abundance of 47.8% (Fig. 5a). Despite direct and mediated electron transfer to electrode was prevented by the nafion coat, bacteria were also found attached to this material, being *Pseudomonas* again the most prevalent genus (52.6%). More detailed information on the composition of the inoculum and the communities developed in the MFC can be found in Additional file 3: Table S3.

### Taxonomy of archaea

Archaea seemed to be less relevant than Bacteria in our experiments. Although a fair amount of diversity was retrieved from the inoculum (4190 sequences grouped into 60 OTUs), only a few of these groups developed in the MFCs. In fact, rarefaction curves confirmed that most of the diversity was retrieved (data not shown). From the three experiments, the MFC-A electrode (biofilm) harbored the most diverse community with 77 OTUs including a high percentage of unique OTUs (49.3% of the sample) in comparison to the anolyte samples (average of 16.6% of unique OTUs per sample). Archaeal community in this sample was composed of the *Crenarchaeota* classes, *Thermoprotei* (27.5%), and *Thaumarchaeota* (41.3%), as well as *Euryarchaeota* class*,* the *Methanomicrobia* (26.8%) (Fig. 5b). Remarkably, neither representatives of the *Thaumarchaeota* nor the *Thermoprotei* classes were found in any of the samples from the anolyte, even in MFC- A. In the case of the *Thaumarchaeota*, the most abundant genus was *Nitrosopomilus*. This genus largely dominated the inoculum sample (85.2% of the sample) but only developed in the MFC-A anode biofilm.

About the suspended communities, the acetoclastic methanogenic family *Methanosaetaceae* covered almost all the archaeal community of the three reactors (about 99%). The most abundant genus, being a 98–99% of the OTUs present in all anolyte samples was affiliated to *Methanosaeta,* a type of acetoclastic methanogen (Fig. 5b).

## Discussion

Understanding how operating conditions influence bacterial community and power density can boost the performance of MFCs. Yet, determining the mechanisms of current production of each microorganism is challenging. Here, we attempted to evaluate the differences in performance of MFCs related to the electron transfer mechanisms carried out by the microorganisms through in-depth electrochemical and microbial composition analyses. Although assigning a functional role to a microbial group based on its taxonomy is not an easy task, the anode modifications of our experimental setup ease at least the formulation of hypotheses about the putative functions of the most abundant groups recovered.

In general, differences in the performance and efficiency among the three MFC experiments were observed. Although in all cases acetate was consumed quickly, which indicates an efficient degradation of the carbon source by microbial communities, overall very low efficiencies were registered. Internal resistances (R_int_) calculated from polarization curves showed high values in all cases (Table 1). Competing microbial processes such as methanogenesis lower MFC efficiency and, thus, energy is lost for electricity production [28]. Nevertheless, no methane was detected in the MFCs headspace. Besides, aerobic degradation of the substrate was prevented by continuously sparging nitrogen. However, other factors related to the reactor design, such as electrode spacing, can also affect coulombic efficiencies [29, 30]. The low efficiency of our MFCs could be caused by the H-shape fuel cell reactor design, as seen previously [31]. Additionally, the degree to which a particular microbial community spends energy on respiration versus growth and biosynthesis [29, 32] is also important. During the experiment, DAPI counts showed an increase in the number of microorganisms in the three reactors, indicating that bacterial growth could also be responsible for the low efficiencies observed in the MFCs. Nevertheless, the present study demonstrates that stable power could be generated through the three known electron transfer mechanisms.

**Fig. 6:**
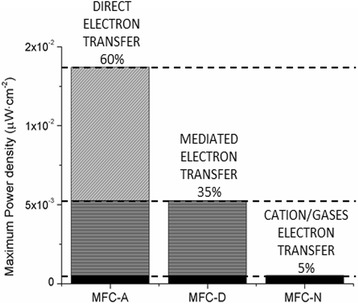
Extrapolation of the results, obtained to each condition reactor, to the MFC-A in which all electron transfer mechanisms were possible

Among the possible electron transfer mechanisms, direct contact was the one with a higher efficiency in power. We estimated the contribution of direct electron transfer mechanism produced in MFC-A by subtracting the values of mediated electron transfer by electron shuttles (measured in MFC-D) and fermentation products (hydrogen) production (measured in MFC-N) from the total power produced in MFC-A (Fig. 6). Results showed that about 60% of the total power output is generated by direct electron transfer and thus, despite the presence of an important community of non-attached electrogenic bacteria in suspension, we can conclude that the biofilm was responsible for most of the current produced (Fig. 6). In order to confirm this observation, the anode electrode of MFC-A was removed and replaced by a new electrode at the end of the experiment. At that time, power density ​​suffered a 70% decrease (from 1.37e^−2^ to 4.38e^−3^ μW·cm^−2^) down to values ​​close to those obtained for MFC-D (an average of 4.99e^−3^ ± 4.08e^−4^ μW·cm^−2^), confirming a higher contribution of the biofilm and, consequently, of direct electron transfer to power generation.

In terms of community composition, sequences related to previously reported electroactive bacteria such as *Shewanella* [7] or *Aeromonas* [25] were found in the biofilm of MFC-A. However, anode voltammogram showed peaks in common with the anolyte, indicating the existence of electron shuttles attached to the biofilm matrix. Species of *Aeromonas* are able to reduce ferric iron, nitrate and sulfate, expressing *c*-type cytochromes when grown under anaerobic conditions with oxidation and reduction peaks at 50 and −350 mV (vs. Ag/AgCl) [25]. The potential of these reported peaks coincides with some of those found in the MFC-A biofilm voltammetry. Other genera found in the biofilm that could be contributing to direct electron transfer include *Anaerovorax* [33] and *Empedobacter* [34].

Members of the genus *Flavobacterium* have previously been found in the anolyte community of microbial electrochemical systems [35], but their role in current production remains unknown in part due to their broad metabolic capabilities. The high contribution of this genus in MFC-A as compared to the other reactors could indicate the capacity of these bacteria to direct electron transfer to an anode. Regarding the genus *Acinetobacter*, some species are known to use a self-secreted redox compound identified as pyrroloquinoline quinone [25, 36]. The fact that members of this genus presented high abundances in the suspended community of MFC-A as well as in the MFC-D reactor, which allowed only mediated electron transfer, suggests that these organisms may play an important role in mediated electricity production. Yet, further experiments are needed to confirm this hypothesis.

In addition, the selection of the archaeal groups *Nitrosopumilus and Thermoprotei* only on the electrode suggests that they could be responsible for direct electron transfer. This hypothesis is supported by the fact that *Thermoprotei* is able to use ferric iron as an electron acceptor [37]. However, a lack of knowledge about electricity production by Archaea prevents us from confirming this hypothesis. This observation, nevertheless, opens up the door to further investigating the role of Archaea in current production in electrosynthesis systems.

About the performance observed by the use of mediated electron transfer mechanisms, a decrease in power of about a 60% related to MFC-A (Fig. 6) was observed attributing a 35% of energy produced to mediated electron transfer by electron shuttles (MFC-D) and a 5% to fermentation products such as hydrogen (MFC-N). Among the community developed using mediated electron transfer by electron shuttles, 50% of the OTUs found were related to *Arcobacter*. Electrochemical activity of members of *Arcobacter* had previously been found attached to the electrode of acetate-fed fuel cells [38]. Despite this could be an indication of their ability to direct transfer, the actual mechanism of electron transfer has not yet been established. Nonetheless, it is known that power output is not only dependent of the cells in contact with the anode but upper layers of the biofilm can also contribute to electron transfer by producing electron shuttles. In this way, its presence in MFC-D points out towards a mediated mechanism for this bacterium.

Finally, the MFC-N showed the lowest values in all parameters as compared to the other MFCs. In this reactor *Pseudomonas* was the most prevalent genus. This genus can produce pyocianine and other phenazine derivates [23, 24] inside the MFC reactors. However, the restriction for electron transfer in this reactor suggests an alternative metabolic pathway of *Pseudomonas* in MFC-N. A feasible possibility for electricity generation, taking into account the conditions within the reactor, would be from biohydrogen [39]. Although this is only a hypothesis the ability of *Pseudomonas* to produce hydrogen has previously been reported under different conditions [40].

The three MFCs contained in suspension the practically unique presence of a single archaeal genus, *Methanosaeta,* able to perform methanogenesis [41]. However, methane was not detected in the headspace of the anode chamber except at the beginning of the experiment and at very low values. On the basis of these results, a possibility would be that *Methanosaeta* was performing a type of metabolism closely resembling syntrophic acetate oxidation, in which the hydrogen consumption prevents *Methanosaeta* from producing methane [42, 43]. In fact, some species of this genus, i.e. *Methanosaeta termophila,* have been shown to produce hydrogen [44, 45]. However, a syntrophic acetate oxidation requires an accompanying organism taking up the reducing power released to the medium as hydrogen. Most of the time the hydrogen-using companion is either a sulfate reducer or a hydrogenotrophic methanogen. In this case, the ability of hydrogen to diffuse through the nafion suggests that hydrogen produced was also oxidized in the anode producing current. Additionally, several studies have demonstrated the capacity of some archaeal genera of accepting electrons through biological electrical connections with some bacteria [46, 47]. More precisely, it has been demonstrated that *Methanosaeta* species are able to exchange electrons via direct interspecies electron transfer in co-cultures with *Geobacter* species for the reduction of carbon dioxide to methane [47]. However, very little is known about the contribution of archaeal groups to current production in MFCs. Although the metabolic roles assigned to our sequences should be interpreted with care, these results provide some hints on the function of Archaea in these systems.

In summary, three MFCs with different electrode configuration were run in order to control the available electron transfer mechanisms. Different communities developed in each reactor revealing the influence of the electron transfer mechanisms. Furthermore, the differences in microbial composition translated in changes in MFC performance. Within these communities, biofilms seem to be the major contributors to current production in MFCs than suspended cells. Despite a direct link between the presence of certain taxa and their contribution to current production cannot be ascertain, characterization of the complex microbial assemblages in these systems can help us unveiling new electrogenic microorganisms and improve our understanding in their role to the functioning of MFCs. These results represent a progress in our understanding of the electron transfer mechanisms in relation to electrochemical performance and microbial community profiles in microbial fuel cells.

## Conclusion

Three MFCs with different electrode configuration were run in order to control the available electron transfer mechanisms. Different communities developed in each reactor revealing the influence of the electron transfer mechanisms. Furthermore, the differences in microbial composition translated in changes in MFC performance. Within these communities, biofilms seem to be the major contributors to current production in MFCs than suspended cells. Characterization of the complex microbial assemblages in these systems can help us unveiling new electrogenic microorganisms and improve our understanding in their role to the functioning of MFCs. These results represent a progress in our understanding of the electron transfer mechanisms in relation to electrochemical performance and microbial community profiles in microbial fuel cells.

## Additional files

Additional file 1: Table S1. Average and standard error of the peak potential of the species redox found along the experiment in the anolyte of the three MFCs by cyclic voltammetry. The gray cells indicate the presence of the redox compound in the reactor. (TIFF 77 kb)

Additional file 2: Table S2. Number of sequences and diferent OTUs found in each sample by 454-pyrosequencing. (TIFF 96 kb)

Additional file 3: Table S3. Relative phylogenetic distribution (%) of OTUs in the three reactors. (TIFF 269 kb)

Additional file 4: Figure S1. Rarefaction curves of bacterial 16S rRNA OTUs defined by 3% sequence variations in the inoculum, anolyte and anode samples based on the Chao1 diversity estimator. (TIFF 550 kb)

## Abbreviations

CLSM: Confocal Laser Scanning Microscopy
DET: Direct Electron Transfer
j: Current density
MET: Mediated Electron Transfer
MFC: Microbial Fuel Cell
OCV: Open Circuit Potential
PBS: Phosphate Buffer Saline
PEM: Proton Exchange Membrane
Rint: Internal resistance
SEM: Scanning Electron Microscopy
SHE: Standard Hydrogen Electrode
U: voltaje
