# Supplementary figures and images for: Electrochemical performance and microbial community profiles in microbial fuel cells in relation to electron transfer mechanisms
Source: BMC Microbiol. 2017 Oct 18;17:208. doi: 10.1186/s12866-017-1115-2 (PMC5648455; doi:10.1186/s12866-017-1115-2)

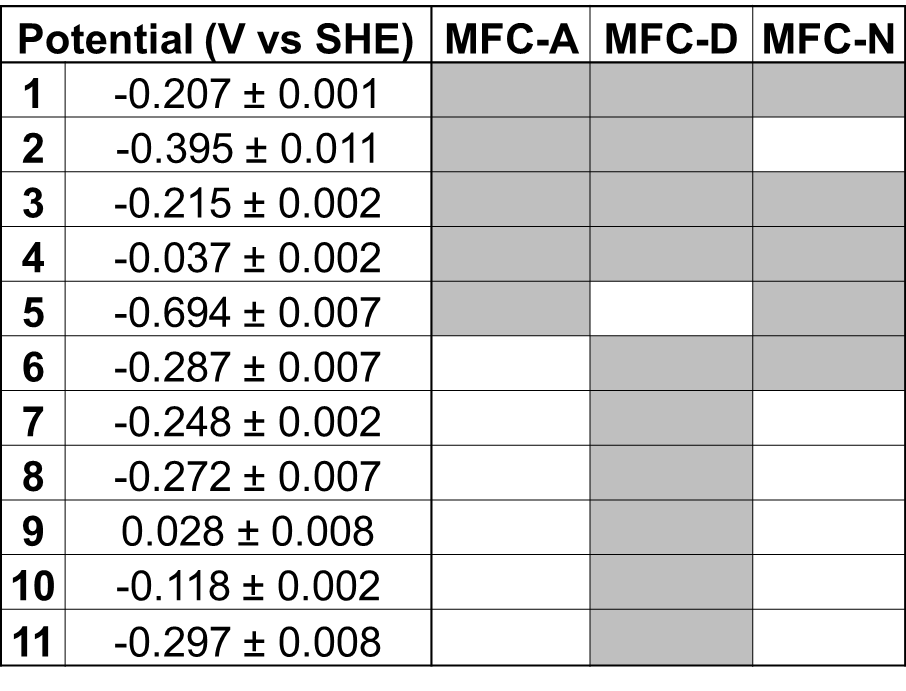

Supplement: Supplementary file 1 — Average and standard error of the peak potential of the species redox found along the experiment in the anolyte of the three MFCs by cyclic voltammetry. The gray cells indicate the presence of the redox compound in the reactor. (TIFF 77 kb) [file 12866_2017_1115_MOESM1_ESM.tif]

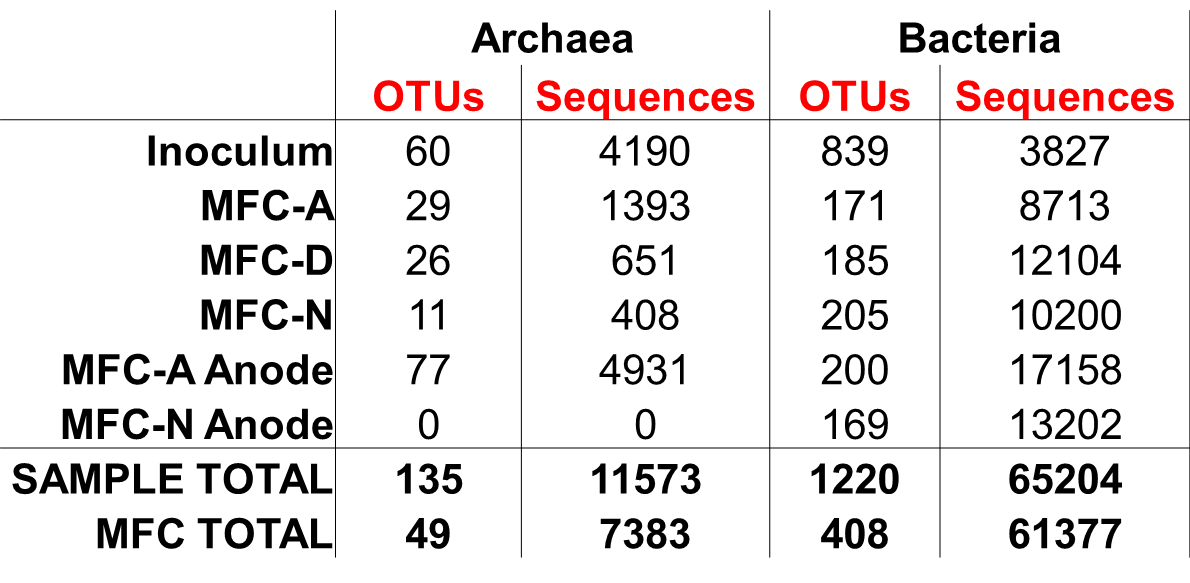

Supplement: Supplementary file 2 — Number of sequences and diferent OTUs found in each sample by 454-pyrosequencing. (TIFF 96 kb) [file 12866_2017_1115_MOESM2_ESM.tif]

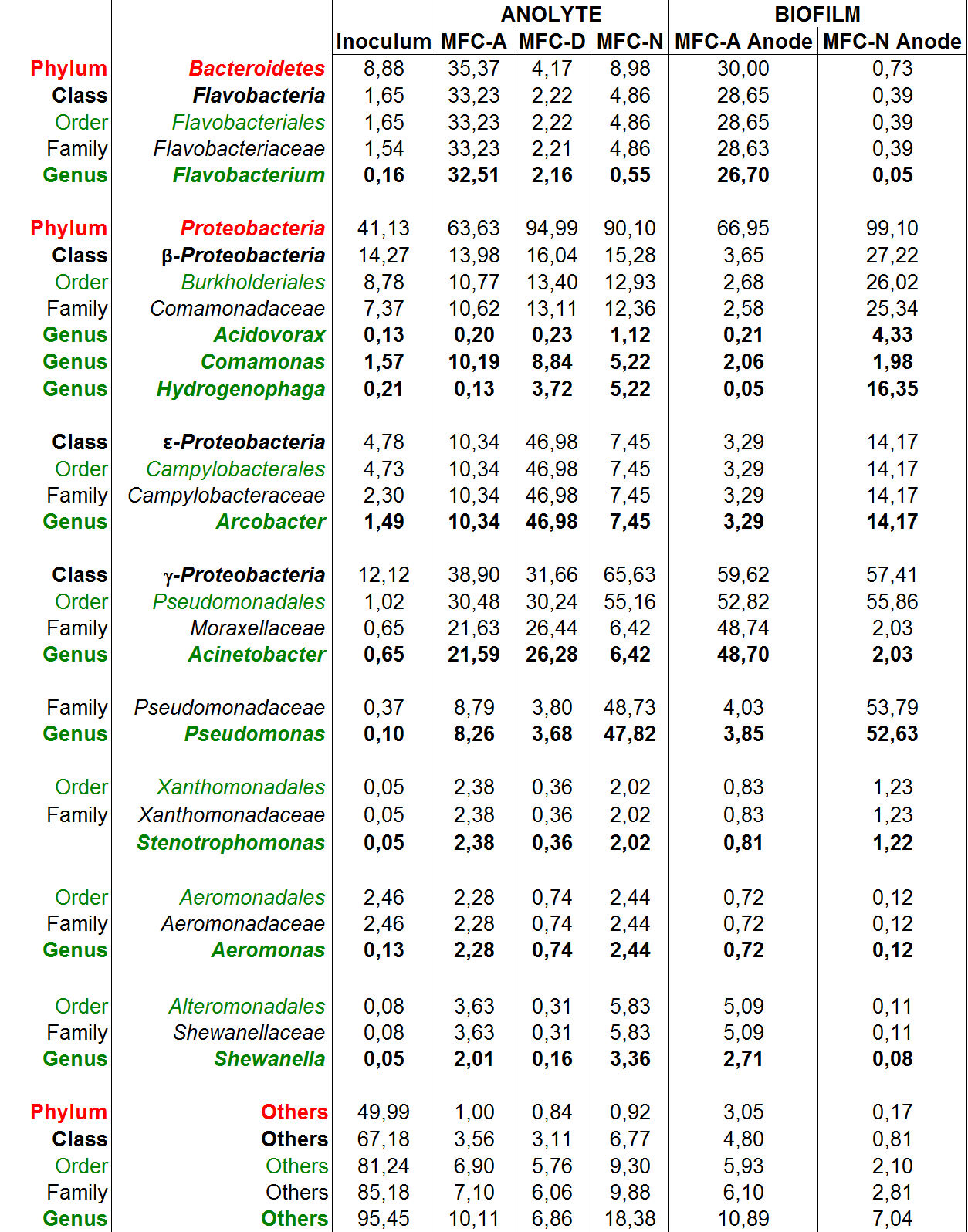

Supplement: Supplementary file 3 — Relative phylogenetic distribution (%) of OTUs in the three reactors. (TIFF 269 kb) [file 12866_2017_1115_MOESM3_ESM.tif]

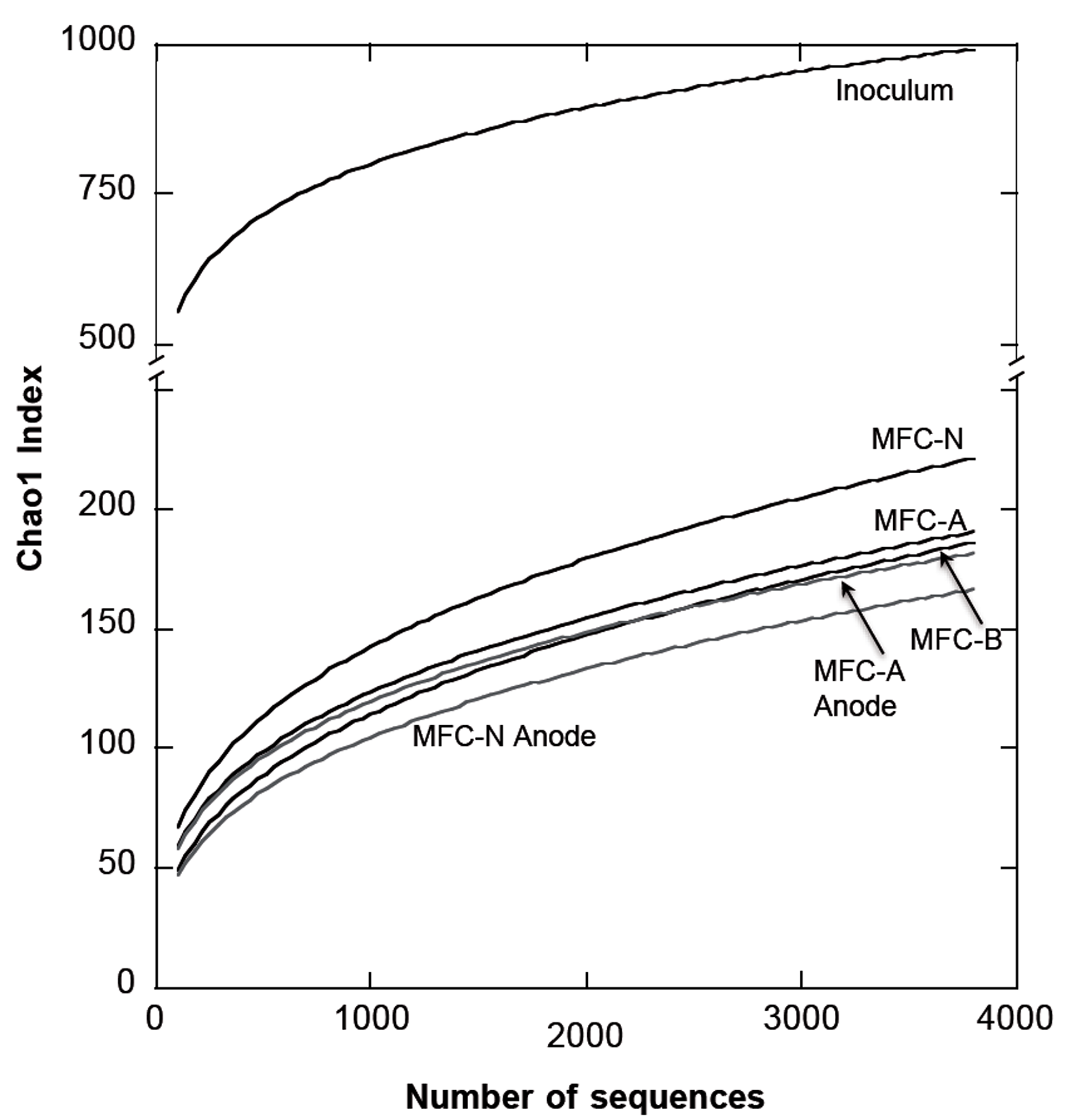

Supplement: Supplementary file 4 — Rarefaction curves of bacterial 16S rRNA OTUs defined by 3% sequence variations in the inoculum, anolyte and anode samples based on the Chao1 diversity estimator. (TIFF 550 kb) [file 12866_2017_1115_MOESM4_ESM.tif]
